# MAIT cells in respiratory viral infections in mouse and human

**DOI:** 10.1615/CritRevImmunol.2021040877

**Published:** 2021

**Authors:** Yuqing Long, Timothy SC Hinks

**Affiliations:** 1 Respiratory Medicine Unit and National Institute for Health Research (NIHR) Oxford Biomedical Research Centre (BRC), Nuffield Department of Medicine Experimental Medicine, University of Oxford, OX3 9DU, Oxfordshire, UK; 2 Chinese Academy of Medical Sciences Oxford Institute (COI), University of Oxford, Oxford, UK

**Keywords:** Mucosal-associated invariant T cell, virus, infection, innate, T cells, human, mouse, review, COVID-19

## Abstract

Mucosal associated invariant T (MAIT) cells were first identified as specific for bacterial, mycobacterial and fungal organisms, which detect microbially-derived biosynthetic ligands presented by MHC-related protein 1 (MR1). More recently two unexpected, additional roles have been identified for these ancient and abundant cells: a TCR-dependent role in tissue repair and a TCR-independent role in antiviral host defence. Data from several classes of viral disease shows their capability for activation by the cytokines interleukin(IL)-12 / -15 / -18 and type I interferon. MAIT cells are abundant at mucosal surfaces, particularly in the lung, and it seems likely a primary reason for their striking evolutionary conservation is an important role in early innate defence against respiratory infections, including both bacteria and viruses. Here we review evidence for their TCR-independent activation, observational human data for their activation in influenza A virus, and *in vivo* murine evidence of their protection against severe influenza A infection, mediated at least partially via IFN-gamma. We then survey evidence emerging from other respiratory viral infections including recent evidence for an important adjuvant role in adenovirus infection, specifically chimpanzee adenoviruses used in recent coronavirus vaccines, and data for strong associations between MAIT cell responses and adverse outcomes from coronavirus-19 (COVID-19) disease. We speculate on potential translational implications of these findings, either using corticosteroids or inhibitory ligands to suppress deleterious MAIT cell responses, or the potential utility of stimulatory MR1 ligands to boost MAIT cell frequencies to enhance innate viral defences.

## Introduction

Mucosal-associated invariant T (MAIT) cells are abundant, evolutionarily conserved, innate-like T cells defined by their semi-invariant αβ T cell receptor (TCR) and restriction by the nonpolymorphic molecule major histocompatibility complex (MHC)-related protein-1 (MR1)(1). MAIT cells were first described in 1999(2) based on their unique TCR. This comprises a semi-invariant TCR-α chain – typically Vα7.2–Jα33/12/20 in humans, and Vα19–Jα33 in mice – associated with a limited repertoire of β-chains: predominantly Vβ2/Vβ13 in humans and Vβ6/Vβ8 in mice(2-6), though with additional Vβ chain usage in some clones(6). A seminal discovery in field was the identification of naturally occurring MR1 ligands, which were found to be small-molecule biosynthetic derivatives of riboflavin (vitamin B2) synthesis(7-9). As this pathway is present in a wide range of prokaryotic cells, but absent from mammals, detection of such ligands provides for highly specific host-pathogen discrimination, and rightly focussed attention on the role of MAIT cells in providing defence against the bacteria, mycobacteria and yeasts (9-13) capable of their synthesis.

**Figure 1:**
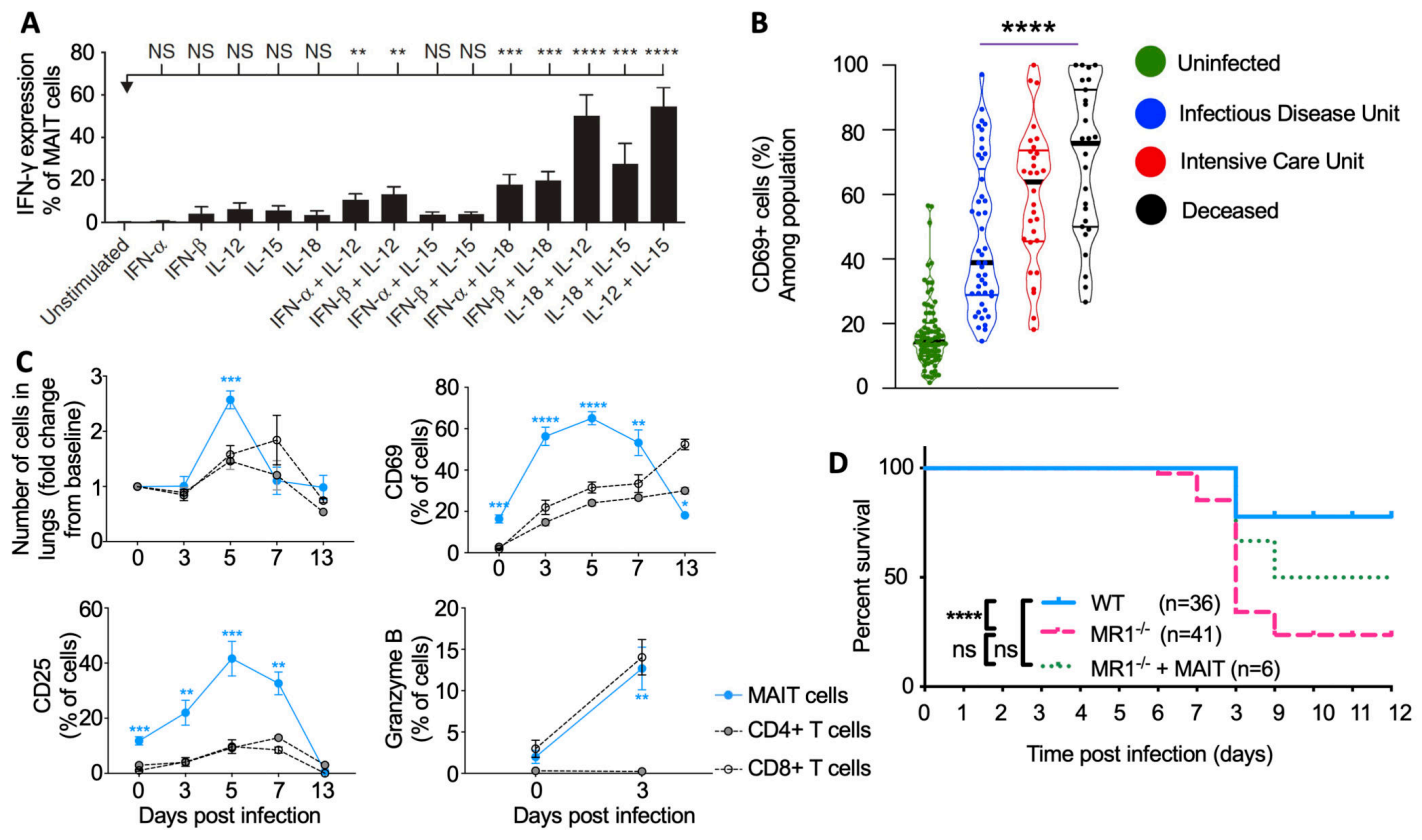
Key published data on MAIT cell activation by influenza A and COVID-19 **A**. MAIT cells respond to interleukins and type I interferons *in vitro*. PBMCs from healthy individuals were directly stimulated for 24 h with IFN-α, IFN-β, IL-12, -15, -18 or combinations thereof. IFN-γ expression by MAIT cells (gated on live CD3^+^CD8^+^CD161^++^Vα7.2^+^ cells) was analysed by flow cytometry. Bars represent means ± S.E.M. Statistical significance was determined with the Kruskal–Wallis test followed by the Dunns’ test. **B**. Human MAIT cell activation in moderate (n=45), severe (n=30) and fatal (n=27) COVID-19, or uninfected controls (n=80). Frequency of CD69+ cells among MAIT cells compared by two-sided Mann–Whitney non-parametric test. **C**. Accumulation and activation of MAIT cells and ‘conventional’ CD4^+^ and CD8^+^ T cells in C57BL/6 mice before and after challenge with 100 plaque-forming units (PFU) influenza A (PR8) virus relative to uninfected day 0 controls at 0, 3, 5, 7, 13 days post-infection. Proportion of pulmonary MAIT cells expressing CD25, CD69 and granzyme B expressed as a percentage of MAIT, CD4^+^ or CD8^+^ T cells. Graphs show combined data (mean ± SEM) from four (days 0, 3, 5), two (day 7) or one (day 13) independent experiments with similar results, with 2–5 mice per group in each replicate. Statistical tests: Kruskal–Wallis one-way ANOVA with *post hoc* Dunn’s tests. **D**. MR1^−/−^ mice show enhanced mortality in response to infection with 100 PFU PR8 virus compared with wild-type mice. Combined data from one (MR1^−/−^ + MAIT cells) to four experiments (WT; MR1^−/−^), compared using log-rank (Mantel–Cox) tests. Figures reproduced from (17)(A), (61)(B) and (34)(C,D) with permission. NS>0.05, *P 0.05, ***P≤0.001, ****P≤0.0001. CD, cluster of differentiation; IFN, interferon; IL, interleukin; MAIT, mucosal associated invariant T; MR1, major histocompatibility complex-related protein-1; NS, non -significant; PMBC, peripheral blood mononuclear cell; WT, wild type.

Unlike conventional T cells, which express highly variable TCRs, capable of targeting a huge breadth of peptide epitopes including those from bacteria, malignant cells and bacteria, the limited diversity of the MAIT cell TCR did not initially suggest a role in anti-viral defence. However several recent studies have revealed at least two additional, entirely distinct activities of MAIT cells. One of these is the TCR-dependent activation of a transcriptional programme, first described in H2M3 restricted Tc17 cells(14), which mediates tissue repair functions and we now know can be activated in human and murine MAIT cells(5, 15, 16). The second additional function of MAIT cell is a TCR-independent mode of activation which can be triggered by virally-induced cytokines which include interleukins (IL)-12, -15, -18 and type I interferon (Figure 1A) (17).

MAIT cells develop in early life from a pool of pre-committed type-17 CD161++CD8αβ + T cells which express promyelocytic leukaemia zinc finger protein (PLZF)(18), and come to dominate the adult CD161++CD8+ T cell population, comprising up to 95% of these cells(19). MAIT cells, like other CD161^++^ CD8^+^ T cells and natural killer (NK) cells, highly express the IL-18 receptor, and were shown *in vitro* to respond to dual stimulation by IL-12 and IL-18 by producing interferon (IFN)-γ(19). This activation follows a slower time course than TCR dependent activation, was of a lower maximal magnitude, and occurred without concomitant production of tumour necrosis factor (TNF) or IL-17A(20). Significantly this IL-12/-18 response could be triggered in peripheral blood mononuclear cells (PMBC) purely by the addition of ssRNA40, a single stranded RNA agonist of the Toll-like receptor TLR8, suggesting a potential for activation by viruses alone(19). This TLR8 agonist activated MAIT cells indirectly by triggering IL-12/-18 production from monocytes(20). *In vitro* cytokine-stimulated MAIT cells upregulated the activation marker CD69, and the cytotoxic serine protease granzyme B, and have been shown also to be triggered by the additional virus-induced cytokine IL-15 and the type I interferons IFN-α and IFN-β, again acting synergistically(17, 21). IL-7 can also activate MAIT cells inducing upregulation of the TCR and CD69, and MAIT cell cytotoxicity(22, 23). This cytokine-induced transcriptional programme is shared with invariant natural killer T (iNKT) cells and is believed to be driven by expression of the master transcription factor PLZF (24, 25).

In 2013 MAIT cells were found to be depleted, activated and functionally exhausted in blood and mucosal tissue during chronic HIV infection(26, 27). The first data from naturally occurring respiratory viral infections were reported by van Wilgenburg *et al* in 2016, showing evidence of peripheral blood MAIT cell activation in participants with acute dengue virus, hepatitis C and influenza A virus (IAV)(17). In dengue MAIT cell activation – measured by CD38 expression – peaked at the day of defervescence, and correlated with more severe disease and with plasma IL-18. In hepatitis C infection correlated with MAIT cell granzyme B expression and cytokine-stimulated MAIT cell supernatants limited viral replication *in vitro*. Similar and upregulation of granzyme B were observed in influenza both *in vivo* and *in vitro*. Together these data strongly implicate MAIT cells in antiviral responses.

MAIT cells are abundant at mucosal surfaces, particularly in the lung, and it seems likely a primary reason for their striking evolutionary conservation is an important role in early innate defence against respiratory viruses. Therefore, here we will review evidence for the role of MAIT cells in responding to respiratory viral infections in humans and *in vivo* evidence of their protection against severe influenza A infection, mediated at least partially via IFN-γ. We survey evidence emerging from other respiratory viral infections, including adenovirus infection, and coronavirus-19 disease (COVID-19), and speculate on potential translational implications of these findings in promoting antiviral defence or limiting immunopathology.

## MAIT cell responses to influenza A virus in humans

Resting human MAIT cells are characterized by a lack of granzyme B and low perforin expression, two key granule proteins required for efficient cytotoxic activity. Instead they express high levels of granzyme A and granzyme K. However MAIT cell activation can rapidly induce granzyme B and perforin, licensing these cells to kill target cells(28, 29). This upregulation of granzyme B and perforin occurs between 6 and 18 hours, with most MAIT cells expressing these molecules by 30 hours(28). Bacteria and mycobacteria can upregulate other cytotoxic molecules such as the antibacterial pore-forming molecule granulysin(30, 31) which can kill intracellular and extracellular bacteria, but this occurs much more slowly(30), and only efficiently disrupts microbial membranes(32), so is less likely to be relevant for antiviral defence. In patients hospitalised with IAV peripheral blood MAIT cell granzyme B expression is significantly elevated compared with healthy, uninfected controls(17), consistent with acquisition of cytolytic effector function. By contrast absolute MAIT cell frequencies are reduced in peripheral blood in IAV infection, particularly in severe disease in those requiring intensive care or who died from IAV(33). Reduced peripheral blood frequencies could represent either activation induced cell death as occurs in HIV infection(26) or recruitment to sites of inflammation, although the latter would be most consistent with murine data(34).

These observational human data were complemented by mechanistic human *in vitro* MAIT cell – antigen presenting cell (APC) co-culture experiments. Co-culture of PBMC for 10 h with an IAV-infected human lung epithelial cell line (A549) induced IFN-γ in MAIT cells, NK cells and γδ T cells, along with granzyme B and CD69 upregulation(33). Consistent with the expectation this would be TCR-independent, responses were not affected by addition of a blocking monoclonal antibody to MR1(17, 33). However, whilst pure populations of NK cells were able to respond directly to virally-infected epithelial cells, pure flow-sorted MAIT cells could not, implying the requirement for a second, accessory cell type(33). Loss of MAIT cell activation after magnetic depletion of CD14+ cells from PBMC(33), or co-culture of MAIT with IAV- or influenza B-infected macrophages(17) confirmed that monocytes or macrophages could serve this accessory cell function. As infected epithelial cell culture supernatants could activate MAIT cells within a PBMC fraction, this implied MAIT cell activation was dependent on soluble mediators, and not cell contact. Indeed blockade of IL-18 reduced MAIT cell IFN-γ production by 50% implying MAIT cell activation was partially attributable to IL-18(33). Further blockade of additional cytokines, either singly or in combination, showed the dominant role of IL-18 in this response to IAV, in contrast to dengue and hepatitis C where IL-12 and IL-15 respectively were also important, likely because IL-12 and -15 were not induced by IAV infection of monocytes(17). Similar activation of MAIT cells with upregulation of CD38 and CD69 has been observed in PBMC culture with H3N2 IAV infection of human nasal epithelial stem/progenitor cells(35). In summary influenza infection is capable of inducing MAIT cells to upregulate antiviral IFN-γ, and cytolytic granzyme B in a TCR-independent manner, requiring production of IL-18 and potentially other mediators from accessory cells including monocytes and macrophages. However the clinical significance of this effect, whether protective, merely incidental, or even detrimental, could not be determined without *in vivo* experimental challenge.

## MAIT cell responses to influenza A virus in mice

**Figure 2:**
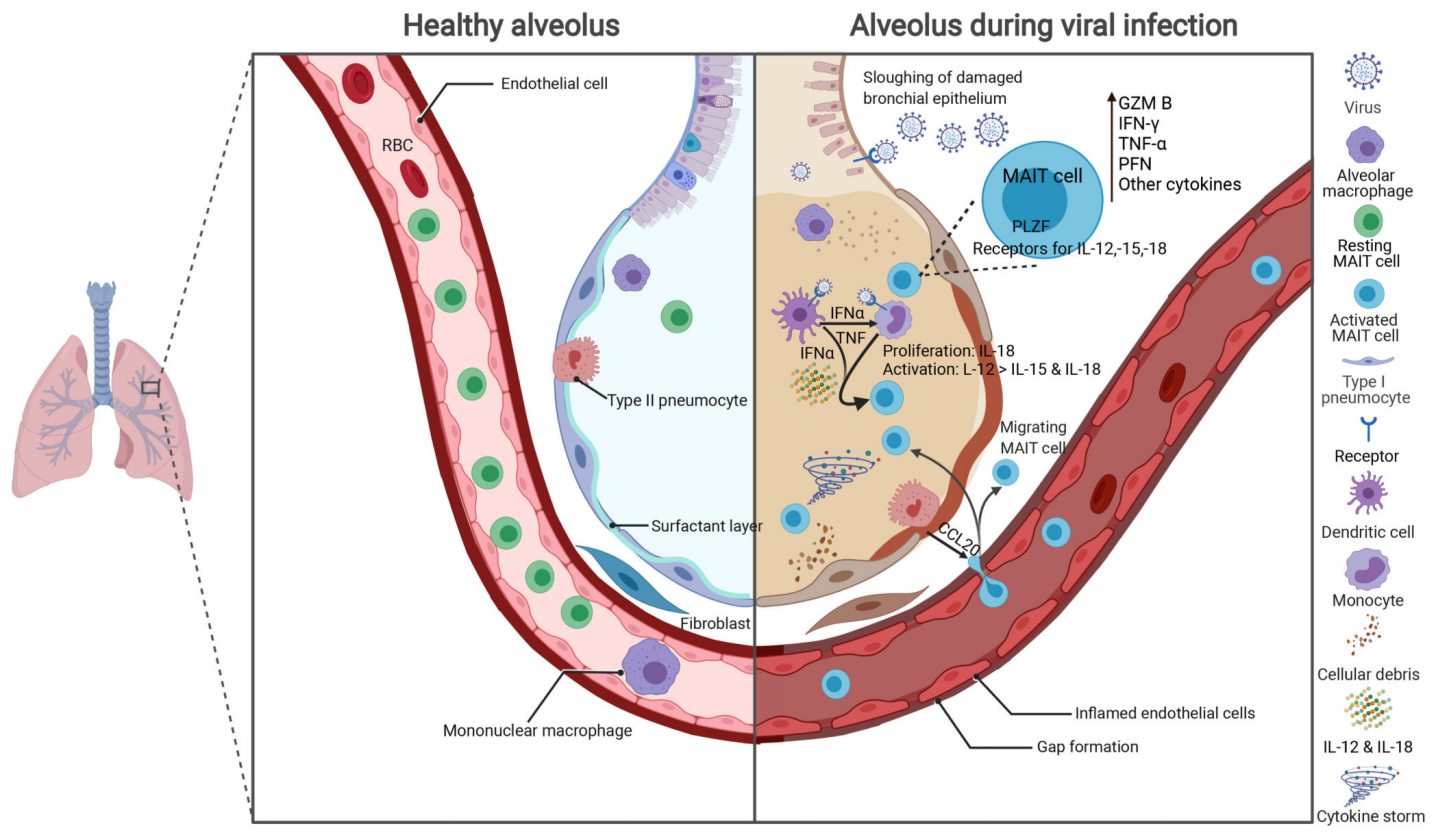
MAIT cells in respiratory viral infections Figure showing a conceptual overview. During homeostasis MAIT cells are present in the circulation, lung parenchyma and alveolus. During infection respiratory viruses invade the bronchial and alveolar epithelia causing release of innate cytokines including type I interferons and alarmins, as well as PAMPs and DAMPs, which lead to activation of DCs and monocytes. Further MAIT cells are recruited from the circulation via chemokines including CCL20. DCs stimulate MAIT cells via type I IFN both directly and indirectly via induction of monocyte cytokine secretion including TNF. Monocytes and other inflammatory cells produce IL-12, -15 and -18 to trigger TCR-independent MAIT cell activation. Activated MAIT cells upregulate inflammatory cytokines including IFN-γ, and TNF, as well as the cytotoxic molecules GzmB and PFN. These changes enhance viral clearance but may contribute to immune pathology including a ‘cytokine storm’ effect. CCL, C-C motif chemokine ligand; DAMP, damage associated molecular pattern; DC, dendritic cell; GzmB, granzyme B; IFN, interferon; IL, interleukin; PAMP, pathogen associated molecular pattern; PFN, perforin; PLZF, promyelocytic leukaemia zinc finger protein; TCR, T cell receptor; TNF, tumour necrosis factor. Created with BioRender.com.

To address this question we challenged C57BL/6 mice with two strains of IAV: the highly pathogenic mouse-adapted influenza virus strain A/Puerto Rico/8/34/1934 (PR8, H1N1), which causes severe pneumonia in mice, and the less pathogenic X-31 (H3N2; A/Hong Kong/X31) strain(34). Consistent with human data, both strains of IAV induced early MAIT cell activation with marked increase of granzyme B expression by day 3 and upregulation of the activation markers CD25 and CD69 peaking at day 5 post infection (Figure 1C). Whilst some activation was also observed in non-MAIT, conventional CD4^+^ or CD8^+^ T cells, activation in MAIT cells was more pronounced and occurred more rapidly. The model allowed us to investigate the mechanism of activation using strains of mice which were deficient in cytokines or cytokine receptors. Pulmonary MAIT cell accumulation was not affected by genetic deficiency of IL-12, IL-15, IFNαR or MR1 but was markedly reduced by deficiency of IL-18. This is interesting because anti-IL-12/-18 blockade did not inhibit TCR-triggered MAIT cell proliferation *in vitro*(28). Conversely MAIT cell activation – measured by CD25 upregulation – was dependent on IL-15, -18 and IFNαR, being markedly reduced in mice lacking each of these pathways, but most notably IL-12, of which deficiency caused a 90% decrease in CD25 upregulation. In contrast deficiencies of these pathways did not affect CD69 expression. Together these findings were consistent with human *in vitro* studies showing complementary but distinct roles of these cytokines in different aspects of MR1-independent, antiviral responses, with dominant roles for IL-18 in pulmonary accumulation, IL-12 and to a lesser extent -15 and -18 in activation, and, at least in human data, type I interferon in upregulation of CD69 – a driver of JAK/STAT signalling (Figure 2).

What might be the consequences of this activation? Activated MAIT cells are potent producers of pro-inflammatory cytokines which have the potential to contribute both to protective host responses or to over-exuberant harmful inflammation, seen in the cytokine storm responsible for approximately half the mortality in severe influenza pneumonia(36). To explore this question we compared weight loss and survival following infection in wild type mice or MR1^-/-^ mice which have an absolute deficiency of MAIT cells, or in MR1^-/-^ mice with a MAIT cell population reconstituted by intravenous adoptive transfer of MAIT cells. We found that with a carefully-titrated inoculum of 100 plaque forming units (PFU) of the highly-pathogenic PR8 IAV there was higher weight loss and mortality in the MR1^-/-^ mice, which was ameliorated by prior MAIT cell adoptive transfer (Figure 1D)(34), confirming an important protective role of MAIT cell antiviral responses.

When exploring the mechanism for this protective effect we did not observe significant differences in viral loads using plaque assays on homogenised lungs. However mortality in this model is more closely linked to the magnitude and quality of the inflammatory response and indeed we found mice lacking MAIT cells had a deficiency SigF^+^CD11b^int^CD64^+^CD11c^+^alveolar macrophages at 3 days post infection associated with reduced total pulmonary T cells numbers by day 5, and evidence of impaired antigen-specific CD8^+^ T cell responses at days 7 and 9. Thus MAIT cells may facilitate and accelerate the evolution of an effective antigen-specific adaptive immune response. This would be analogous to the similar role MAIT cells play in accelerating pulmonary T cell recruitment during intracellular lung infection with the bacterium *Francisella tularensis*, which is mediated by early-MAIT cell induced differentiation of inflammatory monocyte-derived dendritic cells(37). In addition MR1^-/-^ mice had increased total bronchoalveolar lavage protein levels at day 5 post IAV infection, suggesting MAIT cells were protecting against alveolar epithelial damage even relatively early in the time course of the infection(34). Furthermore even by day 3 post infection we observed reduced pulmonary production of IFN-γ and key innate inflammatory cytokines monocyte chemoattractant protein-1 (MCP-1), IL-6 and C-C motif chemokine ligand 5 (CCL5, RANTES) in MR1^-/-^ mice.

As MAIT cells are evolutionarily ancient components of an immune response, as with other innate lymphocyte populations, their functions are obscured by other layers of the immune system, which provide robustness through immunological redundancy(38). For this reason we performed adoptive transfer experiments into immunodeficient Rag2^−/−^γC^−/−^ mice lacking T cell-, B cell- and NK cell-mediated immunity. Whilst such mice ultimately succumb to infection, adoptively-transferred MAIT cells prolonged survival, to a similar extent as transfer of NK cells and more effectively than non-antigen specific CD8 cells. Survival was not prolonged if MAIT cells were generated in IFN-γ^-/-^ mice, demonstrating this protection was, at least partially, dependent on IFN-γ.

These data from influenza studies show that MAIT cells are activated early in a viral infection and contribute to a protective host response, including early innate cytokine production and facilitating development of an adaptive immune response. However many questions remain to be answered, such as how exactly do MAIT cells prolong survival; what role do cytokines other than IFN-γ^-/-^ play; what is the importance of perforin, granzyme and direct cytotoxicity; which cell types are recruited by MAIT cells, through which chemokines and where do these critical events occur – in the lymph node or the tissue?

## MAIT cell responses to other respiratory viruses

To date much less is known about responses to other respiratory viruses. Some interesting data about a potential mechanism of viral MAIT cell activation have been generated using *in vitro* infection with respiratory syncytial virus (RSV), the main cause of respiratory bronchiolitis(39). RSV caused upregulation of the CD161 ligand lectin-like transcript 1 (LLT1) on the respiratory epithelial cell line BEAS-2B and on primary human bronchial epithelial cells. This occurred in infected cells and in uninfected co-cultured cells, suggesting it was induced by soluble factors, which included agonists of TLR3 and TLR2/6, besides type 1 interferons, IL-1 and TNF. The LLT1 accumulated at the site of epithelial cell surface synapses formed with CD161+ T cells. These cells were not specifically identified as MAIT cells, but such a mechanism is very likely to occur with MAIT cells which have high surface CD161 expression. The effect of LLT1-CD161 interactions is poorly understood, and may perhaps protect the epithelium by inhibiting NK cell cytotoxicity and IFN- γ expression(40, 41), but may conversely promote the activation, proliferation and cytokine secretion of T cells(39, 40, 42).

As with IAV, MAIT cells are activated in peripheral blood in humans infected with hantaviruses(43). Hantaviruses are single-stranded negative-sense RNA Bunyaviruses which infect humans who inhale dust containing rodent excreta, leading to two clinical syndromes: hantavirus renal syndrome in Asia and Europe, and hantavirus pulmonary syndrome in the Americas. Pathology involves an excessive immune response with strong release of pro-inflammatory cytokines and highly activated CD8 and NK cells. In people infected with the European-endemic *Puumala orthohantavirus* peripheral blood MAIT cells (but not other lymphocytes subsets) were transiently reduced by 85% during acute haemorrhagic fever, with increased expression of CD69, CD38, and granzyme B in residual circulating MAIT cells(43) over infection days 3-9. High Ki67 expression showed these MAIT cells were proliferating, with Ki67 levels correlating with plasma IL-6. Disease was associated with increased plasma levels of the CCR6 ligand CCL20 and CCR9 ligand CCL25, whilst residual circulating MAIT cells had reduced surface expression of the gut homing maker α4β7 integrin and the mucosal tissue homing marker CCR6, potentially reflecting recruitment of CCR6^+^ MAIT cells to the tissues. *In vitro Puumala orthohantavirus* infection of PBMC caused MAIT cell CD69 upregulation, and infection of MAIT cells in co-culture with the monocyte cell line THP-1 induced upregulation of CD69, CD38 and CD25, as well as the markers of cytolytic capacity perforin, granzyme B and CD107a. This activation was independent of MR1 or of cell-cell contact, but was not induced by inactivated virus and did not induce IFN-γ expression. In the case of hantavirus *in vitro* blocking antibody experiments showed the MAIT cell activation was more dependent on IFN-α than IL-12 or -18.

Measles virus (MeV) poses a much more significant clinical problem, being highly-contagious via respiratory droplets, and remaining a leading global cause of death in infants, with risks of neurological, pulmonary and gastrointestinal complications(44). MeV is a negative strand RNA paramyxomavirus which initially infects nasopharyngeal or conjunctival epithelial cells, then spreading to the regional lymph nodes where it infects lymphocytes and spreads systemically through the lymphoreticular system and generating a secondary viraemia. MeV uses CD46, nectin 4 and CD150 to enter host cells. Whilst nectin 4 is used as the receptor on epithelial cells, CD150 is used to infect immune cells, leading to apoptosis of CD150^+^ memory cells, resulting in immune amnesia(45). Amongst human PBMC and hepatic lymphocytes investigated by single cell sequencing, all cell types expressed CD46 but MAIT cells were the subset with the highest expression of *SLAMF1*, the gene encoding CD150(46). Other innate cells including iNKT and γδT cells, and to a lesser degree central memory and effector memory T cells, also express CD150 highly, though less highly than MAIT cells. Consonant with this expression pattern MAIT cells and iNKT were the most highly infected cell types during *in vitro* infection, and within an hour of infection the majority of MAIT cells stained for Annexin V, a marker of MeV induced apoptosis. This rapid virus-induced apoptosis is likely to contribute to the broad immunosuppression associated with measles, although loss of long-term T cell memory will be related to infection of antigen-specific memory T cells.

Many important respiratory viruses with pandemic potential are zoonoses, including influenza viruses and coronaviruses, which can circulate and evolve in reservoirs including bats. Bat species are long-lived mammals which can carry multiple viruses asymptomatically and are implicated as reservoirs for severe acute respiratory syndrome (SARS), Ebola virus, Nipah virus and SARS coronavirus 2 (SARS-CoV-2). Although only distantly related to humans, MR1 T cells with weak binding to the human MR1-5-OP-RU tetramer, have recently been described at high frequencies in a pteropodid bat, a fruit-eating black flying fox which has strong gene and protein sequence similarity between human and bat MR1 molecules(47). These bat MR1T cells express the classical human MAIT transcription factors PLZF, ROR-γt, T-bet and Eomes and could therefore have an important role in the innate immune defences to these viruses, and would be of interest given the emerging data on MAIT cell responses to SARS-CoV-2, the cause of COVID-19.

## MAIT cells in COVID-19

Recent emergent epidemics of severe acute respiratory syndrome coronavirus SARS-CoV-1 in 2002(48), Middle East respiratory syndrome coronavirus (MERS-CoV) in 2012(49), and the global pandemic of SARS-CoV-2 in 2019 have highlighted the importance to global human health of this group of highly infectious, enveloped, positive-sense single-stranded RNA viruses. The existence of older, endemic alpha- and beta-coronaviruses which now cause milder disease, and the frequent emergence of such new virulent strains implies strong selection pressure on the respiratory immune system, and particularly the value of early innate immune responses to previously unencountered pathogens. This makes it likely that unconventional T cells, including the evolutionarily-conserved MAIT cells contribute to the host response (50, 51).

Since the first reported case in December 2019, in Wuhan, China, of acute respiratory disease caused by SARS-CoV-2 coronavirus disease 2019 (COVID-19)(52) has claimed more than 5 million lives. The different severities and outcomes of COVID-19 vary from mild disease to life-threatening acute respiratory distress syndrome (ARDS) are shaped by the interaction between virus and host antiviral immune responses(53, 54). The key feature of COVID-19 pathogenesis is a dysregulated host immune response, including lymphopenia, neutrophilia, dysregulation of monocytes and macrophages, reduced or delayed type I interferon (IFN-I) response, and even cytokine storm(54-58). Notably, higher plasma levels of pro-inflammatory cytokines or chemokines, such as IL-6, -2, -7, -10, colony stimulating factor (CSF)3 (G-CSF), C-X-C motif chemokine ligand 10 (CXCL10/IP10), C-C motif chemokine ligand 2 (CCL2/MCP1), CCL3 (MIP1A), and TNF and reduced peripheral blood frequencies of lymphocytes, especially T lymphocytes, were found in patients with severe COVID-19 compared to individuals with non-severe disease(54, 57, 59).

Reduced frequencies of MAIT and iNKT, but not γδ T cells are observed in peripheral blood, compared with health or non-COVID critical illness controls(6, 51, 60). In studies of unconventional T cells in blood and airway tissues from patients admitted to intensive therapy unit (ITU) with severe COVID-19, MAIT cell frequencies were higher in paired endotracheal aspirate samples(51, 61) implying the peripheral loss of MAIT cells was likely due to recruitment to the site of inflammation, as would be consistent with MAIT cell downregulation of the tissue-migrating marker CCR6 and the pro-survival marker CD127 in peripheral blood(61). Further evidence for tissue recruitment as the cause of loss of circulating MAIT cells comes from single cell (sc) transcriptomic data of increased chemotaxis-related pathways in peripheral blood MAIT cells in severe COVID-19(62), although in the same analysis there was also found evidence of Caspase-1 activation and pyroptosis, implying more than one process may contribute to MAIT cell decline. Upregulation of CD69 in COVID-19 and non-COVID critical illness(61, 63), and correlation between the presence of MAIT cells and endotracheal CXCL10 and CXCL12 levels, showed these were highly activated unconventional T cells populating the airways of patients, suggesting a potential contribution in the regulation of local inflammation. Circulating MAIT and iNKT cells from COVID-19 patients produced less IFN-γ and higher IL-17A compared with cells from healthy donors. In addition, the plasma levels of IL-18 were higher in COVID-19 patients and positively correlated with the expression of the CD69 activation marker on blood iNKT and MAIT cells, which could be used as a predictor of clinical course and disease severity(51).

A deficiency in peripheral blood MAIT cell frequencies has consistently been reported by others also (60-67), again associated with increased surface expression of activation markers CD38, CD69 and HLA-DR, and with increased production of IL-17, TNF and granzyme B. MAIT cells in COVID-19 patients also upregulated the exhaustion markers CTLA-4 and PD-1(65), as is observed with chronic exposure to viral antigens(68) or inflammation, and associated with hierarchical loss of effector function, followed by activation induced cell death(69). This exhaustion may be driven in part by prolonged exposure to virus-induced IL-12 and -18(70), cytokines which are highly expressed in COVID-19(59). Consistent with this, circulating MAIT cells had significantly lower expression of IL-12 and IL-18 cytokine receptors in comparison to healthy control, which may contribute to the lower expression of IL-12 and -18-induced TNF, perforin and granzyme B in COVID-19(63). There is some evidence *in vitro* this impaired cytotoxicity can be restored by exposure to IL-7(63), a cytokine known to enhance the production of cytolytic molecules in MAIT cells(71). Whilst MAIT cells from COVID-19 patients expressed higher perforin than in health, *ex vivo* expression of IFN-γ, TNF, IL-17A and granzyme B did not differ between these groups, although TCR-induced IL-17 and TNF expression were impaired(65). This impaired MAIT cell effector function in COVID-19 patients may contribute to a reduced capability to control bacterial or viral infection, although it is also possible it could be beneficial in reducing the over-exuberant cytokine production associated with high mortality in COVID-19(58, 72).

Large studies from Oxford(64) and Paris(61) again observed a significant, 90% reduction of circulating MAIT cells in COVID-19, upregulation of granzyme B and CD69 on NK, CD8^+^ T, γδ T and particularly MAIT cells, and confirmed the correlation between the outcome of SARS-CoV-2 infection and MAIT cell activation and cytotoxicity (Figure 1B)(61). Amongst different cell types the highest levels of activation were seen in MAIT cells, particularly in fatal disease. Moreover granzyme B expression was significantly higher in airway rather than peripheral blood MAIT cells, demonstrating marked MAIT cell cytotoxicity in the lungs of patients infected with SARS-CoV-2, and that high MAIT and innate cell activation is predictive of fatal outcomes. Multiparametric matrix correlations showed that in non-surviving patients, blood IL-18 concentrations were strongly positively correlated with MAIT cell granzyme B production. Furthermore peripheral blood IL-18 levels and CD69+ MAIT cell frequencies peaked at days 30-49 of symptoms when the mortality rate was highest.

This MAIT cell activation has been identified as an independent and significant predictor of death in COVID-19, associated with a hazard ratio of 5.9 for mortality and activation of CD8+ T cells and non-Vδ2 γδT cells, and elevated GM-CSF, CXCL10, CCL2, and IL-6(73). In this study it was striking that MAIT cell activation was more associated with fatal outcomes than any other parameter measured, including the clinical prognostic scores SOFA and APACHE II. MAIT cell activation was also associated with mortality in influenza A. Such correlations do not necessarily imply a causative pathological role, but may be a reflection of the sensitivity of MAIT cells to integrate innate antiviral cytokine signals, suggesting that MAIT cell activation is a sensitive, though not a specific, biomarker of disease severity in viral pneumonia

Data from single-cell sequencing of circulating MAIT cells showed further evidence of MAIT cell activation in COVID-19, with the most differential expression of genes in the type I interferon signaling pathway, as well as response to interferon (γ and/or β), regulation of innate immune response, cytokine production, and NF-κB transcription factor activity processes(62). During COVID-19 progression, the cytokine shift from the early type I IFN response to the late IL-18 inflammasome pathway response coordinated by CD14^+^CD16^+^ (intermediate) monocytes correlates with MAIT cell activation(61, 67). MAIT cells in severe COVID-19 patients in intensive care had reduced expression of the type I IFN-mediated gene *IFTIM1*, suggesting in severe disease MAIT cell activation may be mediated by cytokines other than type I IFN, although others have reported increased expression of *IFITIM1, IFTIM2* and *IFTIM3* in less severe COVID-19(62, 74). This suggests perhaps these are important protective pathways, which may be dysregulated in severe disease. Of note, macrophages infected with SARS-CoV-2 upregulate *IFNA, IFNB, IL1B* and *IL6*, and can directly activate MAIT cells *in vitro* and promote the secretion of effectors in a dose-dependent manner(61). Unexpectedly these authors found when blocking MR1 *in vitro*, the ability of infected macrophages to induce cytotoxicity of MAIT cells was dampened, implying that to some extent the activation and induction of MAIT cells are MR1-dependent. This is observation is hard to reconcile with other data on the TCR-independence of MAIT cell antiviral responses, but if replicated could perhaps suggests either a synergy in cytotoxicity by cytokine activated MAIT cells against target cells carrying bacterially-derived MR1 ligand, or perhaps a previously unappreciated role of MR1 in interactions with highly activated MAIT cells. Together, these findings reveal the highly activated phenotype and functional effector production of MAIT cells are associated with disease severity, especially in the context of an IL-18 rich environment.

Some recent data point towards potential mechanisms of suppressed MAIT cell responses in COVID-19. Expression of CD183, CXC3R1, CD8 and CD56 were decreased on peripheral blood MAIT cells in COVID-19, suggesting a functional impairment(74). MAIT cell frequencies and expression of IFN-γ, granzyme B and CD107a were reduced in people with COVID-19/bacterial coinfection, potentially suggesting a functional consequence of this impairment. *In vitro* studies showed that MAIT cell IFN-γ and granzyme B production were impaired by coculture with CD14+ cells from patients with severe COVID-19 and this is mediated by monocyte-derived IL-10 and IFN-α. Several groups have described monocytes with a myeloid-derived suppressor cell-like phenotype in patients with severe COVID-19(75-77), so together these findings suggest myeloid-derived suppressor cell monocytes may mediate functional impairment of MAIT cells through IFN-I-induced IL-10 signalling in severe COVID-19.

A striking feature of SARS-CoV-2 infection is the strong sex differences in morbidity and mortality, with a 1.7-3.4-fold higher risk of death in men than women with COVID-19(78, 79). The explanation for this is unknown, though it has been postulated that sex specific differences in MAIT cell responses could contribute(80). Certainly sex affects very many aspects of the mammalian immune response, and, for instance, it has been reported in COVID-19 that men have higher levels of innate cytokines including IL-8 and IL-18 and stronger activation of non-classical monocytes(81). Such cytokine differences would be expected to affect TCR-independent MAIT cell responses, although the differences in cytokines reported in this study were not statistically significant after adjustments for age and body mass index, and so remain speculative. Furthermore, with large numbers of groups currently exploring correlations between extensive arrays of immunological parameters there is a high risk of type I statistical errors in the field, with any findings requiring prospective validation. With respect to MAIT cells it has been reported that female sex is associated with a more rapid decline in peripheral blood MAIT cells than in males, and that in a *post hoc* analysis of sc Seq data from nasopharyngeal and bronchoalveolar lavage MAIT cells from women there was higher expression of IL-7R, the related signalling genes CISH and SOCS1, and antiapoptotic genes BCL2 and FOXP1(80). However, in these datasets, which did not use tetramers to identify MAIT cells, participant numbers were low, correlations were weak with significant risk of over-fitting models, so caution should be used in interpretation, particularly as the nasopharyngeal dataset included only four female participants(82), and the bronchoalveolar lavage sample only three(83). This paper also identifies two clusters of MAIT cells, one almost exclusively in healthy controls, the other dominating samples from active infection, which likely reflect the signature of virus-induced TCR-independent activation of MAIT cells. Other mechanisms seem more likely for the dramatic sex difference in COVID-19 outcomes, which could include sex-specific differences in expression of the SARS-CoV-2 receptor ACE2, the protease TMPRSS2, FOXP3, IRAK1, type I interferon and a wide range of X-linked immune response genes including IL-4, -10, -13, CD-40L, and TLRs-7 and -8, besides cardiometabolic differences(79).

A second striking feature of COVID-19 is the relationship with obesity with a higher risk of morbidity and mortality(84). Could MAIT cells be relevant? On activation MAITs upregulate glycolytic metabolism, dependent on activation of the metabolic regulator mTORC1, via SLC7A5 amino acid transport, and this is necessary for production of cytokines such as IFN-γ(85). However in obesity MAIT cells are reduced in peripheral blood, increased in adipose tissue, with a more IL-17 dominant phenotype(86) and a reduction in their IFN-γ production(85). It has therefore been speculated that obesity could contribute to dysfunctional MAIT cell responses in COVID-19(87), though such mechanisms might be common to other innate and innate-like cell types, and as with the role of sex, it seems likely many other alternative metabolic factors could also be called on to explain the clinical associations such as vascular endothelial dysfunction(88), altered physiology, comorbidities, chronic-low grade inflammation, hyperinsulinaemia and hyperleptinaemia(88, 89).

In summary, a large body of observational human studies now implicate MAIT cell responses in the immune pathology of COVID-19, with deficiency of circulating MAIT cells, recruitment to the lung, upregulation of activation markers, production of IL-17, TNF and granzyme B, with evidence of MAIT cell exhaustion. SARS-CoV-2 induces the necessary cytokines to activate MAIT cells, and these correlate with MAIT cell activation, but without *in vivo* challenge studies, either murine or human, it remains unclear whether MAIT cells are playing a pathogenic role in severe COVID-19, or a beneficial role in eliminating viral infection(90). Perhaps both could be true, depending on the specific immunology context, with a beneficial role in promoting viral clearance especially at an earlier stage before the development of further severe complications. This speculation is consistent with the proven therapeutic efficacy of the glucocorticoid dexamethasone, which is effective in reducing mortality in severe COVID-19, but is not effective in earlier stage disease: those not requiring respiratory support(91). This seems related to the timing of administration, with two distinct phases of COVID-19: an early phase in which virus pathology dominates and a later phase where immunopathology drives disease(92). The immunosuppressive effects of glucocorticoids at the early stage might hamper antiviral responses, whilst at the later, hyperinflammatory phase of COVID-19 the immunomodulatory effects of glucocorticoids predominate. Again, there are many potential explanations for this, but we have shown that therapeutic glucocorticoids cause rapid loss of circulating MAIT cells(93), an effect which could negatively impact beneficial early antiviral MAIT cell responses, but at later stages could reduce an inappropriate contribution of MAIT cells to the hypercytokinaemia.

## Future Directions

One specific class of respiratory viruses – chimpanzee adenoviruses – has become a recent focus of research interest because of their potential to act as vaccine platforms. This potential has been superbly demonstrated by ChAdOx1 nCoV-19 which expresses the SARS-CoV-2 spike protein and is one of the leading vaccines against SARS-CoV-2 infection(94, 95). Recently the inherent adjuvant activity of MAIT cells has been shown to play an important role in the immunological response to Ad vectors(96). In human PBMC *in vitro* the ChAdOx1 vector strongly activated MAIT cells, inducing dose-dependent upregulation of CD69, granzyme B and IFN-γ. By contrast MAIT cell activation was not induced by the species C vectors Ad5, Ad6 and ChAdN13, which have been shown to poorly stimulate innate immune responses compared with other vectors. Likewise *in vivo* intramuscular ChAdOx1 immunization of C57BL/6J mice and human volunteers also strongly activated MAIT cells. MAIT cell activation in humans correlated with increases in IFN-γ-producing T cells. Gene set enrichment analysis (GSEA) on murine MAIT cells and *in vitro* stimulated human MAIT cells identified similar enrichment of type I IFN, IL-1 family, IL-12 family, and IL-2 family signalling pathways. *In vitro* inhibition of type I IFN, or blockade of IL-18 or IL-12, or depletion of pDC or monocytes, resulted in reduced MAIT cell activation. Therefore, pDC-derived IFN-α and monocyte-derived IL-18 are required in the activation of MAIT cells in response to Ad vectors. Interestingly, IFN-α could also directly induce the production of TNF in monocytes to activate MAIT cells. Mr1^-/-^ mice, which lack MAIT cells, showed defects in the related CD8+ T cell responses when vaccinating the with ChAdOx1 expressing an optimized hepatitis C virus (HCV) antigen, a homologous ChAd63-ovalbumin (OVA) prime-boost immunization and the ChAdOx1-nCoV-19, respectively. In summary, these data suggest the activation of MAIT cells is tightly linked to the immunogenicity of adenovirus vectors and suggest significant potential for harnessing the biological adjuvancy of MAIT cells to enhance the design of vaccines against respiratory viruses.

Research into the antiviral properties of MAIT cells is currently at an early stage and has raised many important questions to address. Firstly, given the potential of MAIT cells to promote viral clearance on the one hand, and to exacerbate immune pathology on the other, with the exception of our *in vivo* data on influenza A, it remains to be determined for any specific virus, and potentially at different stages of disease pathology, whether this balance favours beneficial or protective effects. This can only be determined through *in vivo* studies, which are, for instance, urgently required in SARS-CoV-2 models.

Secondly what are the mechanisms by which MAIT cells effect their beneficial functions in viral infections? MAIT cell-derived IFN-γ certainly contributes(34), but it is not known if this is the only mechanism at play or how this IFN-γ is protective. Does the IFN-γ act directly on target cells to induce an antiviral state, or does it prime other effector cells? If so which are the critical effector cell types and is the IFN-γ acting to promote their recruitment, or cytokine production, or other cytolytic effects? Does IFN-γ act very locally, within an immunological synapse, or more diffusely within a tissue? And what role does the cytolytic capacity of MAIT cells play in viral responses? Might their expression of NK receptors facilitate detection of infected cells? Furthermore the unexpected observation of MR1 dependence of virus-induced MAIT cell cytotoxicity(61) needs to be confirmed and the mechanism elucidated.

Thirdly what role might the recently discovered tissue repair function of MAIT cells (5, 15, 16) play in recovery of the lung parenchyma after a viral pneumonitis?

Fourth, how can the capacities of this ubiquitous, multi-functional cell type be harnessed for clinical translation? Already we have seen the potency of MAIT cells to enhance the immunogenicity of adenoviral vectors, but can their adjuvancy be stimulated against other viral diseases also? Can the use of highly-specific MAIT cell ligands be used to enhance vaccine responses by activating and expanding MAIT cells during immunisation? Can MAIT cell ligands – perhaps in an inhaled format – be safely used to induce an enhanced antiviral state, potentially providing protection or early post-exposure prophylaxis towards a wide range of future emerging viral threats? And in what inflammatory conditions might there be therapeutic potential in using inhibitory MAIT cell ligands to suppress inappropriate MAIT cell activation?

Whilst it will take time and concerted effort to answer such questions as these, the tenor of recent discoveries makes it likely such efforts will be rewarded.

## Abbreviations

5-OE-RU: 5-(2-oxoethylideneamino)-6-D-ribitylaminouracil
5-OP-RU: 5-(2-oxopropylideneamino)-6-D-ribitylaminouracil
COVID-19: coronavirus-19 disease
CSF: colony stimulating factor
GSEA: gene set enrichment analysis
IAV: influenza A virus
IFN: interferon
IL: interleukin
iNKT: invariant natural killer T
MAIT: mucosal associated invariant T
MHC: major histocompatibility complex
MERS: Middle East respiratory syndrome
MR1: major histocompatibility complex-related protein-1
NK: natural killer
SARS-CoV-2: severe acute respiratory syndrome coronavirus 2
TCR: T cell receptor
TLR: Toll-like receptor
